# Pd/MnO_2_:Pd/C Electrocatalysts for Efficient Hydrogen and Oxygen Electrode Reactions in AEMFCs

**DOI:** 10.3390/nano16010071

**Published:** 2026-01-04

**Authors:** Ivan Cruz-Reyes, Balter Trujillo-Navarrete, Moisés Israel Salazar-Gastélum, José Roberto Flores-Hernández, Tatiana Romero-Castañón, Rosa María Félix-Navarro

**Affiliations:** 1 Tecnológico Nacional de México/Instituto Tecnológico de Tijuana, Centro de Graduados e Investigación en Química, Boulevard Alberto Limón Padilla S/N, Mesa de Otay, Tijuana 22000, Baja California, Mexico; ivan.cruz19@tectijuana.edu.mx (I.C.-R.); moises.salazar@tectijuana.edu.mx (M.I.S.-G.); 2 Instituto Nacional de Electricidad y Energías Limpias (INEEL), Avenida Reforma 113, Colonia Palmira, Cuernavaca 62490, Morelos, Mexicotromero@ineel.mx (T.R.-C.)

**Keywords:** Pd/MnO_2_, carbon Vulcan, HOR, anode, AEMFC

## Abstract

Developing cost-effective and durable electrocatalysts is essential for advancing anion exchange membrane fuel cells (AEMFCs). This work evaluates Pd-based catalysts supported on β-MnO_2_, Vulcan carbon (C), and their physical blend (Pd/MnO_2_:Pd/C) as bifunctional electrodes for the oxygen reduction reaction (ORR) and hydrogen oxidation reaction (HOR). The catalysts were synthesized via chemical reduction and characterized by TGA, ICP-OES, TEM, BET, and XRD. Rotating disk electrode studies revealed that the hybrid exhibited superior activity and kinetics, with lower Tafel slopes and higher exchange current densities compared to the individual supports. In AEMFCs, the hybrid reached 128.0 mW cm^−2^ as a cathode and 221.7 mW cm^−2^ as an anode, outperforming individual components. This enhanced performance arises from the synergistic interaction between Pd nanoparticles and MnO_2_, where MnO_2_ modulates the catalyst’s microstructure and local reaction environment while the carbon phase ensures efficient electron transport. MnO_2_, although inactive for the HOR alone, acted as a structural spacer, enhancing mass transport and stability. Durability tests confirmed that the hybrid electrocatalyst retained over 99% of its initial activity after 3000 cycles. These results highlight the hybrid Pd/MnO_2_:Pd/C as a promising, bifunctional, and durable electrocatalyst for AEMFC applications.

## 1. Introduction

Global warming, fossil fuel depletion, and increasing environmental concerns have intensified the need for alternative energy systems that are both sustainable and environmentally friendly. Renewable energy sources such as solar, wind, hydro, and geothermal have gained significant attention as viable solutions to meet global energy demands [1,2,3]. In this context, hydrogen has emerged as a clean and efficient energy carrier that can be produced from renewable sources and used in diverse energy conversion technologies [4,5].

Among hydrogen-based technologies, fuel cells (FCs) are highly efficient electrochemical devices that convert chemical energy directly into electricity. A typical fuel cell consists of an anode and a cathode separated by an electrolyte, and the HOR occurs at the anode and the ORR at the cathode. These electrochemical processes are catalyzed by materials supported on gas diffusion layers or a membrane, and their kinetics greatly influence the overall performance of the device.

In alkaline media, the ORR proceeds via two possible pathways [6]:

(4e^−^ electron pathway)
  (1) $$O_{2}+2\;H_{2}O+4e^{-}\to 4\;{OH}^{-};\;E^{0}=+0.401\;V\;vs.\;SHE$$
(2e^−^ electron pathway)
  (2) $$O_{2}+H_{2}O+2e^{-}\to {HO}_{2}^{-}+{OH}^{-};\;E^{0}=-0.076\;V\;vs.\;SHE$$

The four-electron pathway is preferred because the formation of peroxide species in the two-electron process can degrade the catalyst and membrane, reducing the efficiency and durability of the fuel cell.

The HOR in alkaline media typically follows either a Tafel–Volmer or a Heyrovsky–Volmer mechanism, involving the following elementary steps [7]:

(3) $$H_{2}+2^{*}\to 2\;H_{ad}$$

(4) $$H_{2}+{OH}^{-}+*\to H_{ad}+H_{2}O+e^{-}$$

(5) $$H_{ad}+{OH}^{-}\to H_{2}O+e^{-}+*$$

These reactions can be studied by rotating disk electrode (RDE) voltammetry, where polarization curves typically exhibit kinetic, mixed, and diffusion-limited regions that offer insights into the mechanisms of electron transfer and mass transport limitations in the catalyst layer.

While platinum-based materials are the benchmark electrocatalysts for both the HOR and ORR due to their excellent activity and durability, their high cost and scarcity remain major drawbacks. Although alkaline media enable the use of low-PGM or PGM-free catalysts, particularly at the cathode [8], the HOR remains highly dependent on Pt because of its unique ability to dissociatively adsorb hydrogen even at room temperature [9]. In this context, palladium has emerged as a promising alternative to Pt, owing to its relatively good HOR and ORR activity and lower cost, although its kinetics and stability still lag behind those of Pt, especially in alkaline media [10]. Early studies also demonstrated that the electrocatalytic performance of Pd in alkaline environments can be significantly enhanced through strong metal–support interactions, particularly when combined with metal oxides, providing improved activity and durability compared to conventional Pd/C systems [11]. Nevertheless, emerging non-noble catalysts have also shown promising activity in alkaline environments [12].

In response to these limitations, metal oxides have emerged as promising components in electrocatalyst design, not only for their intrinsic activity but also for their contribution to catalyst stability. Oxides such as TiO_2_, CeO_2_, and MnO_2_ can act as corrosion-resistant supports, electronic modulators, or physical spacers, thereby enhancing long-term catalyst durability and performance [13]. Recent studies have shown that metal–oxide composites (e.g., Ni_4_Mo/TiO_2_) improve HOR performance under fuel cell conditions through strong metal support interactions that stabilize the electronic structure and inhibit catalyst degradation [14]. 

Among transition metal oxides, manganese dioxide (MnO_2_) stands out for its low cost and favorable ORR activity in alkaline environments. However, its poor electrical conductivity remains a major limitation for practical application [15,16,17,18]. Strategies such as morphology control [17,18], cationic doping [19], and defect engineering [20,21] have been used to overcome this issue. Another common approach is to combine MnO_2_ with conductive carbon materials such as carbon nanotubes, graphene, or Vulcan carbon (C), which significantly enhance electron transport [20,22].

Although several strategies have been reported to overcome the intrinsically low electrical conductivity of MnO_2_, such as carbon coating, chemical anchoring, or in situ growth on conductive supports, these approaches often involve complex synthesis routes and limited scalability. In contrast, a physical blending strategy offers a simple and modular alternative that preserves the intrinsic properties of each component. In this configuration, the carbon phase ensures efficient electron transport and high dispersion of Pd nanoparticles, while MnO_2_ contributes catalytic functionality and structural stability, acting as a redox-active and corrosion-resistant spacer within the electrode.

Furthermore, hybrid materials composed of MnO_2_ and noble metals such as Pd have shown synergistic behavior, enhancing both catalytic activity and stability. For example, Pd-decorated MnO_2_ nanorods have demonstrated improved ORR performance in alkaline media and promising durability in AEMFCs, although their limited electronic conductivity can restrict full utilization of the active surface [21].

In view of the ongoing search for efficient and cost-effective electrocatalysts for alkaline fuel cells, this study explores two complementary systems: Pd nanoparticles supported on MnO_2_ and on Vulcan carbon. While MnO_2_ provides catalytic contribution, its inherently low conductivity hampers electron transport. Conversely, Vulcan carbon ensures excellent conductivity. To combine the strengths of both materials, we propose a simple yet effective strategy: a physical blend of Pd/MnO_2_ and Pd/C, forming a bifunctional hybrid catalyst that integrates the catalytic function of MnO_2_ with the conductive network provided by carbon. This approach is designed to overcome the conductivity limitations of MnO_2_ while preserving and potentially enhancing its catalytic contribution through interfacial synergy.

Accordingly, this study systematically investigates the electrochemical performance of the individual and hybrid catalysts toward both the HOR and ORR using RDE measurements, and further evaluates their viability as anode and cathode materials in AEMFCs. Particular attention is given to the dual role of MnO_2_ as a catalytically active and structurally stabilizing phase in composite electrocatalysts under alkaline conditions.

## 2. Materials and Methods

### 2.1. Materials and Chemicals

Manganese sulfate monohydrate (MnSO_4_·H_2_O, 98%) was obtained from Monterrey Analytic Reagents (Monterrey, México). Potassium permanganate (KMnO_4_, 98%), sodium tetrachloropalladate (Na_2_PdCl_4_, 98%), sodium borohydride (NaBH_4_, 99%), and Nafion^®^ 117 solution (5 wt%) were purchased from Sigma-Aldrich (Darmstadt, Germany). Sodium hydroxide (NaOH, 98%) was supplied by Fermont (Monterrey, México). Ethanol (C_2_H_5_OH, 99%) and methanol (CH_3_OH, 99%) were obtained from Faga Lab (Sinaloa, México). Ultra-high-purity hydrogen (99.999%) and high-purity oxygen (>99.5%) were supplied by Infra^®^ (Hidalgo, México). The anion exchange membrane (AF1-HNN8-25-X, Aemion™, Vancouver, BC, Canada) was used as the separator between anode and cathode. Commercial carbon black Vulcan XC-72 (C) was purchased from the Fuel Cell Store. All reagents were used as received without further purification. Milli-Q^®^ water (18.2 MΩ·cm, Millipore^®^) was used for all aqueous solution preparations.

### 2.2. Synthesis of Pd/MnO_2_ and Pd/C

MnO_2_ was synthesized via a hydrothermal method. An aqueous solution containing 1 mM MnSO_4_ and 1 mM KMnO_4_ (30 mL total) was magnetically stirred for 30 min, then transferred into a stainless-steel autoclave and heated at 120 °C for 12 h in a convection oven (Memmert, UF110Plus, Schwabach, Germany). The resulting precipitate was washed with deionized water and ethanol, dried overnight at 60 °C, and subsequently calcined in air at 400 °C for 4 h (heating rate: 5 °C min^−1^).

Pd nanoparticles were deposited on MnO_2_ and C supports via chemical reduction. In a typical synthesis, 20 mg of MnO_2_ or C were dispersed in 50 mL of methanol under magnetic stirring for 15 min. A solution of Na_2_PdCl_4_ (5.43 mM in methanol) was added dropwise to obtain a nominal Pd loading of 10 wt%. After reduction, the materials were washed with methanol and water, then dried at 60 °C overnight.

### 2.3. Physicochemical Characterization

Inductively coupled plasma–optical emission spectroscopy (ICP-OES, PerkinElmer^®^ Optima 8300, Syngistix Software, Waltham, MA, USA) was used to quantify Pd content in the catalysts. Samples were digested in a 1:3 (*v*/*v*) HNO_3_:HCl solution after thermal treatment at 120 °C for 2 h. Analyses were performed in axial mode using gas, auxiliary, and nebulizer flows of 15.00, 0.20, and 0.55 L min^−1^, respectively, with a radio frequency power of 1300 W. Detection wavelengths were 340.5 nm for Pd and 403.1 nm for Mn. All measurements were performed in quadruplicate.

Transmission electron microscopy (TEM) was used to analyze the morphology and particle size of the catalysts (JEOL^®^ JEM-2200FS, Tokyo, Japan, operated at 200 kV in STEM mode). N_2_ adsorption–desorption isotherms were recorded using a Quantachrome^®^ Autosorb IQ analyzer (Anton Paar Quanta Tec, Boynton Beach, Florida, USA) to determine specific surface area (SSA), pore volume, and pore diameter using the BET method. Crystalline phases were identified by X-ray diffraction (XRD) using a Bruker^®^ D8 Advance diffractometer (Billerica, MA, USA) with Cu Kα radiation (λ = 1.541 Å). Patterns were recorded in the 2θ range of 15–80° with a step size of 0.016° and step time of 2 s.

### 2.4. Electrochemical Characterization

The electrocatalysts were first evaluated using a three-electrode configuration with an RDE (glassy carbon, 0.196 cm^2^) as the working electrode, a Hg/HgO reference electrode (1.0 M NaOH), and a Pt wire as the counter electrode. All potentials were converted to the reversible hydrogen electrode (RHE) scale.

Catalyst inks were prepared by dispersing 2.0 mg of Pd/MnO_2_ or Pd/C in 550 μL ethanol and 150 μL Nafion^®^ solution (5 wt%). For the blended Pd/MnO_2_:Pd/C catalyst, the two inks were mixed in a 40:60 *v*/*v* ratio. A 40 μL aliquot of each ink was deposited on the RDE surface and dried at room temperature. This composition was selected based on a preliminary screening of different Pd/MnO_2_:Pd/C ratios (50:50, 40:60, 30:70, and 20:80), evaluated toward both ORR and HOR in 0.1 M NaOH using RDE measurements. The corresponding polarization curves and comparative discussion are provided in the Supplementary Information (Figure S1). Among the tested compositions, the 40:60 ratio exhibited the highest current densities and the most favorable onset potentials for both reactions, and was therefore chosen as the optimal formulation for subsequent electrochemical and fuel cell studies.

ORR and HOR measurements were carried out in O_2_ or H_2_ saturated 0.1 M NaOH under hydrodynamic conditions at various rotation speeds (100–1600 rpm). Prior to each test, the electrolyte was purged with the respective gas for 20 min.

The CO stripping was performed on a vitreous carbon electrode (0.079 cm^2^) modified with 20 μL of catalytic ink. Both measurements were made in 0.1 M NaOH solution. For CO stripping, the solution was first saturated with CO and a potential of 0.1 V vs. RHE was applied for 15 min to adsorb the CO. Argon was then bubbled for 10 min to remove the CO from the solution. Finally, CO removal was performed by cyclic voltammetry at 50 mV s^−1^.

Stability studies were conducted in a half-cell configuration through accelerated durability tests (ADT), which involved applying 3000 cycles of cyclic voltammetry. Before and after this procedure, linear sweep voltammetry curves were recorded to assess the catalytic activity toward the ORR or the HOR. The specific activity of the catalyst was determined by measuring the current density at 0.5 V vs. RHE for the ORR and at 0.2 V vs. RHE for the HOR. These studies were performed using a Biologic SP-150 potentiostat/galvanostat (EC-Lab^®^, Nashville, TN, USA).

### 2.5. Membrane Electrode Assemblies (MEA) Preparation

MEA were prepared by depositing the catalyst ink onto the gas diffusion layers (GDL) to fabricate the gas diffusion electrodes (GDE). The total palladium loading was maintained at 0.5 mg cm^−2^ for both the anode and cathode. The catalyst ink was formulated by dispersing a Pd/MnO_2_:Pd/C hybrid (in a 40:60 weight ratio), Pd/C or Pd/MnO_2_ in a mixture of deionized water, ethanol, and AEMION+^®^ 825 ionomer.

For comparative purposes, commercial Pt/C was used as the counter electrode in selected MEA. Prior to assembly, both the membrane and the GDE were hydrated in deionized water for 24 h, followed by ion exchange in 1.0 M NaOH for 48 h to convert the membrane to the hydroxide (OH^−^) form.

### 2.6. Fuel Cell Testing Protocol

The electrochemical performance of the MEA was evaluated in a single-cell AEMFC test station (Scribner^®^ 850, Scribner Associates, Inc, Carolina del Norte, USA) equipped with humidification units and temperature control. The active geometric area of the MEA was 5.0 cm^2^. The anode and cathode were fed with ultra-high-purity hydrogen and oxygen, respectively, at flow rates of 0.125 and 0.250 L min^−1^ and a backpressure of 10 psia was applied to both sides. Humidifiers were set to 60 °C, matching the cell operating temperature, to ensure fully humidified gases.

The MEA were assembled using Teflon™ gaskets and graphite flow field plates with serpentine channels. A torque of 107 in·lbf was applied to ensure uniform compression. Prior to performance testing, MEA were conditioned under open circuit for 1 h at 60 °C with gas flows on both sides, followed by a 30 min polarization at 0.3 V to stabilize the interface. All measurements were conducted in potentiodynamic mode by scanning from open circuit potential (OCP) down to 0.3 V at a rate of 25 mV s^−1^. Polarization and power density curves were collected after stabilization, and the data reported correspond to the average of at least three independent runs using fresh MEA.

## 3. Results and Discussion

### 3.1. Physicochemical and Morphological Characterization

Inductively coupled plasma–optical emission spectroscopy (ICP-OES) was employed, yielding a Pd content of 9.60 ± 0.4 wt% for Pd/MnO_2_ and 9.50 ± 0.7 wt% for Pd/C. The close similarity in metal content among all synthesized catalysts, including the physical blend Pd/MnO_2_:Pd/C, which exhibited a nominal 10.0 ± 0.5 wt% Pd, demonstrates a high degree of control and reproducibility in the synthesis protocol.

Beyond metal loading, the specific surface area and porosity of the materials are critical factors influencing electrocatalytic activity, as they determine the accessibility and distribution of active sites. These textural properties were evaluated from N_2_ adsorption–desorption isotherms, and surface areas were calculated using the Brunauer–Emmett–Teller (BET) method.

As listed in Table 1, Pd/C showed the highest surface area (51.50 m^2^ g^−1^), attributed to the porous nature of Vulcan carbon. In contrast, Pd/MnO_2_ exhibited a significantly lower value (16.10 m^2^ g^−1^), reflecting the more compact, crystalline nature of MnO_2_. Interestingly, the physical mixture Pd/MnO_2_:Pd/C showed a surface area of 37.01 m^2^ g^−1^, closely matching the theoretical average between the two pure components. This indicates that the mixing process preserved the native morphology and surface properties of the individual phases without significant structural compaction or obstruction of porosity. The observed deviation between experimental and theoretical surface areas was less than ± 0.88%, further supporting the effective blending of both components.

The average pore size of all samples remained in the range of 1.76–1.80 nm, showing minimal variation and suggesting a consistent mesopore regime across the materials. However, differences in pore volume were more pronounced. Pd/MnO_2_ exhibited the lowest pore volume (9.58 × 10^−3^ cm^3^ g^−1^), while Pd/C reached the highest value (3.26 × 10^−2^ cm^3^ g^−1^), consistent with the expected structural properties of the respective supports. The intermediate pore volume of the blended material reflects the additive nature of the textural characteristics.

**Figure 1:**
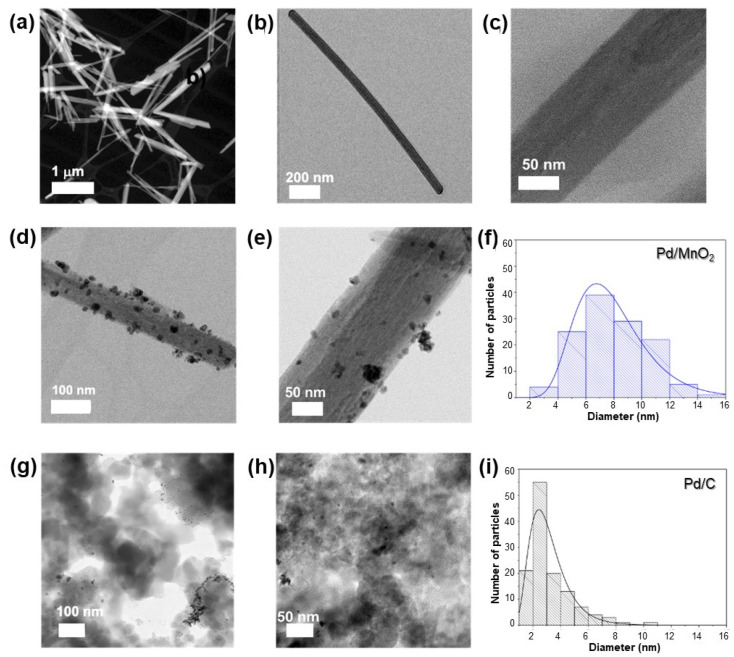
(**a**–**c**) TEM micrographs of MnO_2_ at different magnifications, (**d**,**e**) Pd/MnO_2_ and (**g**,**h**) Pd/C. Histograms of (**f**) Pd nanoparticles on MnO_2_ and (**i**) palladium nanoparticles on carbon.

The morphology and particle size distribution of the materials were examined by transmission electron microscopy (TEM). As shown in Figure 1a–c, MnO_2_ exhibits a nanorod morphology with diameters of approximately 100 ± 20 nm and lengths of 1300 ± 170 nm. Pd nanoparticles are uniformly dispersed along the oxide surface (Figure 1d,e), with an average size of 7.9 ± 2.4 nm (Figure 1f). In the Pd/C electrocatalyst (Figure 1g,h), Pd nanoparticles are also well distributed but display some degree of agglomeration, with a significantly smaller average size (3.2 ± 1.6 nm, Figure 1).

These morphological differences highlight the strong influence of the support’s physicochemical characteristics, such as surface area, porosity, and surface chemistry, on Pd nucleation and growth. The carbon support, with its high surface area and abundant anchoring sites, favors the formation of smaller, more uniformly dispersed Pd nanoparticles. This, in turn, can impact the accessibility of active sites and influence the overall electrocatalytic performance.

**Figure 2:**
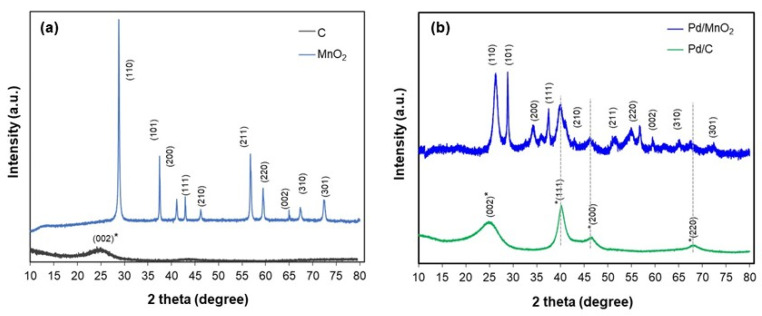
XRD pattern of (**a**) C and MnO_2_ and (**b**) Pd/C and Pd/MnO_2_.

X-ray diffraction (XRD) analysis performed to determine the crystalline phases present in the supports and synthesized electrocatalysts. As shown in Figure 2a, the MnO_2_ support exhibits a series of sharp, well-defined peaks at 2θ = 28°, 37°, 42°, 57°, 60°, and 73°, corresponding to the (110), (101), (111), (211), (002), and (301) planes of the tetragonal β-MnO_2_ phase (space group P4_2_/mnm, JCPDS No. 15-44101). These features indicate high phase purity and crystallinity.

In contrast, the Vulcan XC-72 carbon displays a broad diffraction signal centered around 2θ ≈ 24°, attributed to the (002) plane of graphitic carbon (JCPDS No. 01-074-2329). The broad and low-intensity nature of this peak is consistent with the turbostratic structure of amorphous carbon black.

Figure 2b shows the XRD patterns of the Pd/MnO_2_ and Pd/C catalysts. Both exhibit characteristic reflections of face-centered cubic (fcc) metallic palladium at approximately 2θ = 40.1°, 46.5°, and 86.0°, corresponding to the (111), (200), and (220) planes (space group Fm-3m, JCPDS No. 05-0681). This confirms the successful formation of crystalline Pd nanoparticles, with no detectable oxide or secondary phases.

A slight shift in the Pd reflections toward lower 2θ values (by ~0.2–0.5°) is observed in the Pd/MnO_2_ sample relative to Pd/C. This shift may indicate lattice expansion in Pd, possibly arising from interfacial strain, size effects, or partial incorporation of interstitial species during nucleation on MnO_2_. Although alloying is not expected, the result suggests that the oxide support subtly alters the local structural environment of Pd.

### 3.2. Electrochemical Characterization

#### 3.2.1. Oxygen Reduction Reaction

**Figure 3:**
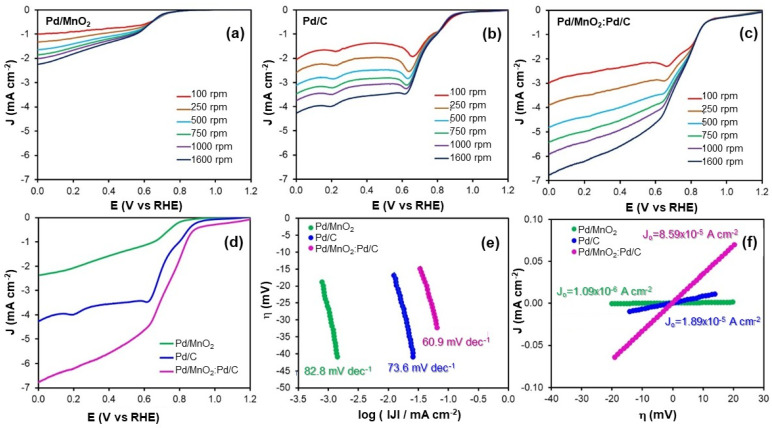
(**a**–**c**) Linear sweep voltammograms recorded at different rotation rates in O_2_-saturated 0.1 M NaOH for: (**a**) Pd/MnO_2_, (**b**) Pd/C, and (**c**) Pd/MnO_2_:Pd/C. (**d**) Comparison of polarization curves for the three catalysts at 1600 rpm. (**e**) Tafel plots obtained from the kinetic region. (**f**) Overpotential (η) versus current density (J) plots at low overpotentials.

Figure 3a–c shows the polarization curves recorded at various rotation speeds for the ORR on the evaluated electrocatalysts. None of the catalysts exhibit a well-defined diffusion-limited current plateau, even at high rotation rates, indicating the absence of a purely mass transport-controlled regime. This behavior suggests that the ORR proceeds under mixed kinetic–mass transport control within the investigated potential range. Although increasing the rotation speed enhances oxygen transport from the bulk electrolyte, additional limitations such as charge transfer kinetics and oxygen diffusion within the catalyst layer remain significant. Similar behavior has been reported for Pd-based and oxide-containing electrocatalysts, where the multistep nature of the ORR in alkaline media, combined with heterogeneous active sites and internal mass transport limitations within porous catalyst layers, prevents the establishment of an ideal diffusion-limited regime, even at high rotation rates [6].

Figure 3d presents the polarization curves obtained at 1600 rpm. In the absence of a clearly defined diffusion-limited region, it was not possible to accurately determine either the diffusion-limited current density or the half-wave potential. To evaluate catalytic activity under kinetic control, the current density at a fixed potential of 0.5 V vs. RHE was selected as a performance indicator. At this potential, the Pd/MnO_2_ catalyst delivered a current density of −1.15 mA cm^−2^, while Pd/C achieved −3.52 mA cm^−2^. Notably, the hybrid Pd/MnO_2_:Pd/C catalyst demonstrated the highest activity, reaching −5.17 mA cm^−2^.

Additionally, the potential required to attain a current density of −2 mA cm^−2^ further highlights the catalytic differences. Pd/MnO_2_ required only 0.11 V vs. RHE, while Pd/C and Pd/MnO_2_:Pd/C needed 0.71 V and 0.79 V, respectively. Although Pd/MnO_2_ shows lower overall ORR activity, these results confirm that the hybrid Pd/MnO_2_:Pd/C outperforms both individual components and operates more efficiently at lower overpotentials.

To further investigate the reaction kinetics, Tafel analysis was performed. The Tafel slope was derived from the linearized form of the Butler–Volmer equation (Equation (6)):

(6) $$\eta =a+bLog(J)\;\;\;b=\frac{2.303RT}{\alpha nF}$$

where *η* is the activation overpotential (mV), *R* is the universal gas constant (8.314 J mol^−1^ K^−1^), *T* is the temperature (K), *α* is the charge transfer coefficient, *n* is the number of electrons in the rate-determining step, *F* is the Faraday constant (96,485 C mol^−1^), *J* is the current density (mA cm^−2^), and *J*_0_ is the exchange current density. The slopes were obtained from the linear region of the *Log*(*J*) vs. *η* plots (Figure 3e), and the results are summarized in Table 2.

Among the three electrocatalysts, Pd/MnO_2_:Pd/C exhibited the lowest Tafel slope (60.9 mV dec^−1^), followed by Pd/C (73.6 mV dec^−1^) and Pd/MnO_2_ (82.8 mV dec^−1^). A slope near 60 mV dec^−1^ is typically associated with a first-electron transfer as the rate-determining step, assuming ideal adsorption conditions. Therefore, the hybrid catalyst demonstrates more favorable ORR kinetics, requiring lower overpotentials to drive the reaction.

The exchange current density (*J*_0_), a key parameter representing intrinsic catalytic activity under near-equilibrium conditions, followed the same trend. The hybrid Pd/MnO_2_:Pd/C catalyst exhibited the highest *J*_0_ (8.59 × 10^−5^ mA cm^−2^), significantly outperforming Pd/C (1.89 × 10^−5^ mA cm^−2^) and Pd/MnO_2_ (1.09 × 10^−6^ mA cm^−2^). These values, obtained using the low-overpotential approximation of Equation (7), support the superior charge transfer properties and intrinsic activity of the hybrid system.

(7) $$\eta =\frac{RT}{J_{0}F}\;J$$

These findings underscore a synergistic interaction between the redox-active MnO_2_ and the conductive carbon supports. While MnO_2_ facilitates oxygen adsorption and stabilization of intermediates, the carbon matrix promotes efficient electron transport and Pd dispersion. Consequently, the hybrid Pd/MnO_2_:Pd/C catalyst achieves enhanced ORR performance, even under kinetic control and in the absence of a diffusion-limited regime.

Although a true limiting current was not attained, the electrochemical data remain highly relevant for practical applications, as fuel cells often operate under kinetic constraints. The improved behavior of the hybrid electrocatalyst highlights its potential for further optimization and implementation as a cathode material in alkaline fuel cells. Future efforts may focus on tailoring the morphology and porosity of the catalyst layer to mitigate mass transport limitations and enhance overall performance.

#### 3.2.2. Hydrogen Oxidation Reaction

The HOR, which occurs at the anode, is a fundamental process in hydrogen fuel cells and is typically more dependent on Pt-based catalysts than the ORR at the cathode. In alkaline media, its kinetics are significantly slower compared to acidic environments, owing to additional factors such as hydroxide adsorption and the complexity of proton-coupled electron transfer steps [23]. Analysis of the Tafel slope provides valuable mechanistic insight by revealing both the reaction kinetics and the nature of the rate-determining step.

In general, a Tafel slope of approximately 30 mV dec^−1^ has been associated with the Tafel step (dissociative adsorption of hydrogen) being rate-limiting, particularly in acidic media. However, in alkaline media, this interpretation is not always straightforward. Slopes in the range of 30 to 120 mV dec^−1^ may indicate different controlling steps: a slope near 120 mV dec^−1^ is typically linked to the Volmer (proton discharge) or Heyrovsky (electrochemical desorption) steps (Equations (4) and (5)), depending on the nature of the catalyst and the adsorption properties of the surface [24]. These variations reflect the influence of the electrolyte environment and the surface chemistry of the electrocatalyst.

**Figure 4:**
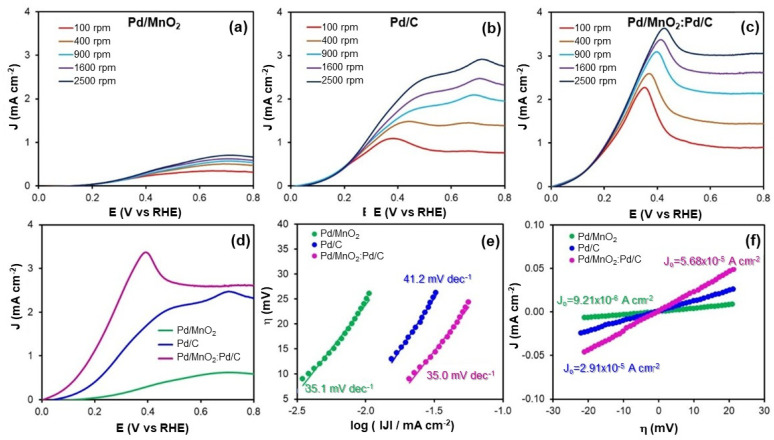
(**a**–**c**) Linear sweep voltammograms recorded at different rotation rates in H_2_-saturated 0.1 M NaOH for: (**a**) Pd/MnO_2_, (**b**) Pd/C, and (**c**) Pd/MnO_2_:Pd/C. (**d**) Comparison of polarization curves for the three catalysts at 1600 rpm. (**e**) Tafel plots obtained from the kinetic region. (**f**) Overpotential (η) versus current density (J) plots at low overpotentials.

Figure 4a–d shows the polarization curves for HOR obtained in H_2_-saturated 0.1 M NaOH for the three electrocatalysts under study. These curves provide comparative information on the kinetic behavior of the materials and reveal differences in catalytic efficiency under anodic conditions.

The kinetic behavior of the electrocatalysts toward the HOR was further evaluated by analyzing their Tafel slopes (Figure 4e and Table 3). The measured values were 35.1 mV dec^−1^ for Pd/MnO_2_, 41.2 mV dec^−1^ for Pd/C, and 35.0 mV dec^−1^ for the hybrid Pd/MnO_2_:Pd/C. These slopes are close to the theoretical value of 30 mV dec^−1^, which is typically associated with the Volmer–Tafel mechanism. In this pathway, the HOR proceeds via the dissociative adsorption of hydrogen molecules (Volmer step), followed by the chemical recombination or desorption of adsorbed hydrogen atoms (Tafel step), with the latter acting as the rate-determining step.

The slightly lower Tafel slopes observed for the hybrid Pd/MnO_2_:Pd/C (35.0 mV dec^−1^) and Pd/MnO_2_ (35.1 mV dec^−1^) compared to Pd/C (41.2 mV dec^−1^) suggest more favorable HOR kinetics. This improvement may be attributed to enhanced hydrogen adsorption and recombination at the Pd/MnO_2_ interface or to electronic modifications induced by MnO_2_ on the Pd sites. In contrast, the higher slope for Pd/C could indicate a less efficient surface process, potentially due to suboptimal hydrogen binding energy or limited surface coverage.

These findings confirm that all three catalysts follow a Volmer–Tafel mechanism under alkaline conditions. The hybrid Pd/MnO_2_:Pd/C catalyst benefits from a synergistic combination of MnO_2_ and carbon support, which together facilitate improved charge transfer and hydrogen activation, resulting in enhanced HOR kinetics compared to the individual components.

Table 3 also summarizes the electrokinetic parameters, including Tafel slopes and exchange current densities (*J*_0_) for the HOR. The exchange current density is a key kinetic descriptor of the intrinsic electrocatalytic activity near equilibrium; higher *J*_0_ values reflect faster charge transfer and lower activation barriers for the reaction.

In this study, the *J*_0_ values determined for the HOR were 9.21 × 10^−6^ A cm^−2^ for Pd/MnO_2_, 2.91 × 10^−5^ A cm^−2^ for Pd/C, and 5.68 × 10^−5^ A cm^−2^ for the hybrid Pd/MnO_2_:Pd/C. These results clearly demonstrate an enhancement in intrinsic activity upon combining MnO_2_ with the carbon-based support.

The hybrid catalyst exhibited the highest J_0_ value, nearly twice that of Pd/C and more than six times that of Pd/MnO_2_. This enhancement is attributed to the synergistic interaction between MnO_2_ and the conductive carbon matrix, which jointly optimize the electronic environment of Pd active sites and facilitate more efficient hydrogen adsorption and oxidation.

The relatively low *J*_0_ of Pd/MnO_2_ highlights its limited HOR activity, likely due to poor electrical conductivity and less favorable hydrogen binding. Pd/C offers improved performance owing to its higher surface area and conductivity. However, the superior *J*_0_ achieved by the hybrid Pd/MnO_2_:Pd/C catalyst illustrates how combining oxide and carbon supports can substantially improve the catalytic performance beyond what either component offers individually.

#### 3.2.3. Electrochemical Active Surface Area (ECSA)

**Figure 5:**
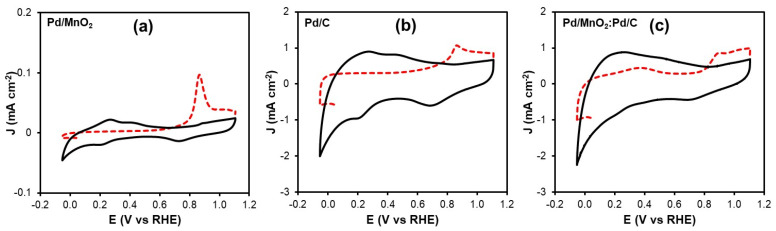
Cyclic voltammetry by CO stripping: (**a**) Pd/MnO_2_, (**b**) Pd/C and (**c**) Pd/MnO_2_:Pd/C.

The electrochemical active surface area (ECSA) of the Pd-based catalysts was determined by CO stripping voltammetry, a reliable technique for quantifying the number of electrochemically accessible Pd active sites. Figure 5a–c shows the CO stripping voltammograms obtained for Pd/MnO_2_, Pd/C and the Pd/MnO_2_:Pd/C blend. In all cases, the first anodic sweep (highlighted in red) corresponds to the electrooxidation of adsorbed CO species, while the second sweep confirms the complete removal of CO from the Pd surface after the initial scan.

A well-defined CO oxidation peak is observed at approximately 0.88 V vs. RHE for all catalysts. This peak is associated with the electrochemical oxidation of adsorbed CO according to the following reaction [25]:

(8) $${\mathrm{CO}}_{\mathrm{ad}}+{\mathrm{OH}}_{\mathrm{ad}}+{\mathrm{OH}}^{-}\;\to \;{\mathrm{CO}}_{2}+e^{-}+H_{2}O$$

which is characteristic of Pd-based electrocatalysts in alkaline media. The charge associated with the CO oxidation peak (*Q*_CO_) was used to calculate the *ECSA* of *Pd* (*ECSA_Pd_*) using Equation (9), assuming a charge density of 0.420 mC cm^−2^ for a monolayer of CO adsorbed on *Pd*.

(9) $${ECSA}_{Pd}=\frac{Q_{\mathrm{CO}}}{0.420\;mC\;{cm}^{-2}\;L_{Pd}}$$

Table 4 summarizes the *Q*_CO_ and *ECSA_Pd_* values obtained for the different catalysts. The pristine Pd/MnO_2_ catalyst exhibits the lowest *ECSA_Pd_* value (3.40 m^2^ g^−1^), indicating a limited number of electrochemically accessible Pd sites, which is consistent with its inferior ORR and HOR performance. In contrast, Pd/C shows a higher *ECSA_Pd_* of 13.37 m^2^ g^−1^, reflecting the beneficial role of the conductive carbon support in enhancing Pd dispersion. Notably, the Pd/MnO_2_:Pd/C blend displays the highest *ECSA_Pd_* value (20.17 m^2^ g^−1^), which is significantly higher than that of both single-component catalysts. This increase suggests that combining Pd/MnO_2_ with Pd/C promotes a more effective utilization of Pd active sites, likely due to improved dispersion, enhanced electronic interactions at the Pd–MnO_2_ interface, and facilitated charge transport through the carbon support. The higher *ECSA_Pd_* of the blended catalyst provides a strong structural basis for its superior electrocatalytic activity toward both ORR and HOR observed in the polarization measurements.

Based on the ECSA values obtained from CO stripping, the intrinsic catalytic activity of the materials was further assessed by normalizing the current density to the electrochemically active Pd surface. For the ORR, intrinsic activities were calculated using the current density measured at 0.2 V vs. RHE, yielding values of −5.3 × 10^1^, −4.9 × 10^2^, and −1.19 × 10^3^ A g^−1^ of Pd for Pd/MnO_2_, Pd/C, and Pd/MnO_2_:Pd/C, respectively. Similarly, HOR intrinsic activities were determined at 0.3 V vs. RHE, resulting in 4.4, 1.39 × 10^2^, and 4.70 × 10^2^ A gPd^−1^ for Pd/MnO_2_, Pd/C, and the hybrid catalyst. These results clearly demonstrate that the superior ORR and HOR performance of the Pd/MnO_2_:Pd/C catalyst arises not only from its higher ECSA, but also from a more efficient utilization of Pd active sites, consistent with the kinetic trends discussed in Section 3.2.1 and Section 3.2.2.

#### 3.2.4. Accelerated Durability Test (ADT)

**Figure 6:**
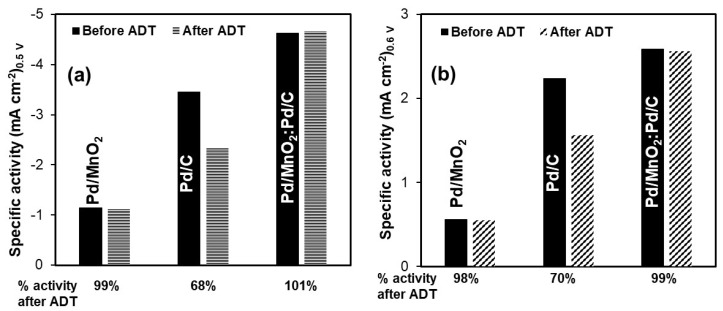
Stability results of the Pd/MnO_2_, Pd/C, and hybrid Pd/MnO_2_:Pd/C electrocatalysts for (**a**) the oxygen reduction reaction and (b) the hydrogen oxidation reaction. Measurements were performed at 1600 rpm in 0.1 M NaOH, saturated with O_2_ and H_2_, respectively.

The stability of the Pd-based electrocatalysts toward both the ORR and the HOR was evaluated using ADT, and the results are summarized in Figure 6. For the ORR (Figure 5a), Pd/MnO_2_ retained 99% of its initial specific activity after ADT, indicating excellent structural stability despite its relatively low intrinsic activity. Pd/C, although initially more active, exhibited significant degradation, retaining only 68% of its original activity. In contrast, the hybrid Pd/MnO_2_:Pd/C catalyst not only showed the highest initial activity but also demonstrated outstanding durability, fully retaining 100% of its activity after ADT.

A similar trend was observed for the HOR (Figure 5b). Pd/MnO_2_ again displayed high durability, preserving 98% of its activity, although its initial performance remained limited. Pd/C showed noticeable degradation, maintaining only 70% of its original activity. Remarkably, the hybrid Pd/MnO_2_:Pd/C catalyst achieved both the highest activity and excellent stability, retaining 99% of its activity after the accelerated test.

It is important to note that these ADT experiments were performed under a three-electrode configuration in a half-cell setup using a RDE, rather than under full AEMFC operating conditions. While the observed stability trends are encouraging, further long-term testing under actual fuel cell conditions is necessary to fully validate the catalyst’s durability.

Although post-mortem structural characterization (e.g., SEM or XRD after ADT) was not performed in this study, the electrochemical durability trends observed here provide strong evidence of catalyst stability under alkaline conditions. The excellent retention of ORR and HOR activity for the Pd/MnO_2_:Pd/C hybrid suggests that the incorporation of MnO_2_ contributes to improved catalyst layer stability and enhanced utilization of Pd active sites through combined structural and interfacial effects. While the proposed role of MnO_2_ as a structural spacer and interfacial promoter is supported by textural, electrochemical, and durability trends, direct characterization of parameters such as ionomer distribution, wettability, or post-cycling morphology was beyond the scope of this work. These aspects will be the focus of future complementary studies.

### 3.3. AEMFC Performance Test

**Figure 7:**
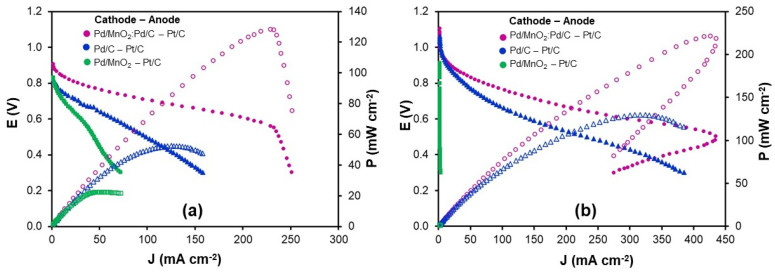
Polarization and power density curves of AEMFCs using the Pd/MnO_2_:Pd/C hybrid, Pd/C and Pd/MnO_2_ electrocatalyst as (**a**) the anode and (**b**) the cathode. Measurements were conducted at 60 °C with H_2_/O_2_ flow rates of 0.125 and 0.250 L min^−1^, respectively. The counter electrode was Pt/C with a loading of 0.5 mg cm^−2^ at both the anode and cathode, as specified.

The electrocatalysts were evaluated in AEMFCs operated at 60 °C using H_2_ and O_2_ as reactants, with 10 psia backpressure applied to both compartments. In the cathodic configuration (Figure 7a), Pd-based catalysts were employed as cathode materials, while commercial Pt/C (0.5 mg cm^−2^) served as the reference anode. The polarization curves revealed the characteristic regions of fuel cell operation: an initial activation region dominated by kinetic limitations, followed by an ohmic region defined by linear voltage loss due to ionic and electronic resistances, and finally a concentration region, where mass transport constraints (such as gas diffusion or water accumulation) prevail [26].

Under these conditions, Pd/MnO_2_ showed the lowest catalytic activity, delivering 30.4 mA cm^−2^ at 0.6 V and a peak power density of 22.4 mW cm^−2^. Pd/C exhibited improved performance, reaching 65.5 mA cm^−2^ and 52.6 mW cm^−2^, respectively. The hybrid Pd/MnO_2_:Pd/C catalyst outperformed both single-component systems, attaining 205.4 mA cm^−2^ at 0.6 V and a peak power density of 128.0 mW cm^−2^. A noticeable decline in current density below 0.5 V suggests the onset of mass transport limitations.

In the anodic configuration (Figure 7b), Pd-based catalysts were evaluated at the anode, with Pt/C serving as the cathode. As anticipated from half-cell results, Pd/MnO_2_ exhibited negligible HOR activity. Pd/C, in contrast, demonstrated robust anodic performance, reaching 143.4 mA cm^−2^ at 0.6 V and 129.0 mW cm^−2^ in power density, with minimal mass transport constraints. The hybrid Pd/MnO_2_:Pd/C catalyst delivered the highest output, achieving 296.3 mA cm^−2^ and 221.7 mW cm^−2^. However, a performance drop at lower voltages was observed, which is likely associated with localized water accumulation in the anode compartment. Such water accumulation effects are known to hinder gas transport and active site accessibility in AEMFC anodes, particularly under high current density operation [27,28].

While MnO_2_ does not significantly contribute to electrocatalytic activity, its presence plays a key structural role in the hybrid system. Acting as a morphological spacer, MnO_2_ promotes an open and porous electrode architecture that facilitates gas diffusion and ionic transport. Nevertheless, under high current densities and in the absence of active anode water removal, this structural contribution alone is insufficient to fully suppress flooding. The water accumulation observed at low voltages is therefore attributed to high reaction rates rather than to limitations in electrode design.

This interpretation aligns with previous reports showing that non-catalytic porous materials, such as graphene nanofibers or activated carbon, can enhance fuel cell performance by modulating internal electrode porosity, enabling efficient water redistribution, and preserving catalytic accessibility [29]. In this context, MnO_2_ within the hybrid structure serves a dual function: as a morphological modulator at the anode, where water generation is prominent, and as a secondary catalytic phase at the cathode, where water is consumed and mass transport conditions are more favorable.

In summary, the Pd/MnO_2_:Pd/C hybrid catalyst demonstrated outstanding bifunctional performance, surpassing Pd/C by a factor of 1.7 and outperforming Pd/C and Pd/MnO_2_ by 2.4 and 5.7 times, respectively, in cathodic operation. These results emphasize the synergy between catalytic activity and structural engineering, validating the hybrid approach as a versatile and effective strategy for dual-electrode applications in AEMFC systems.

**Figure 8:**
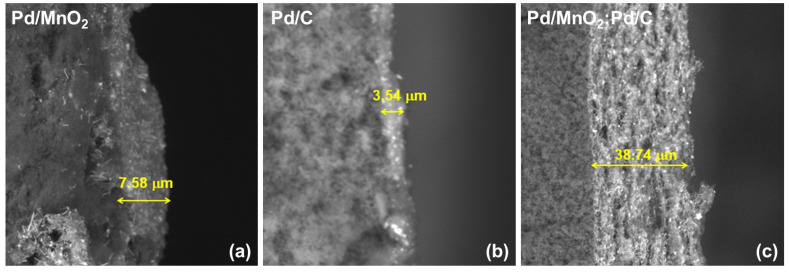
Cross-sectional SEM micrographs showing the thickness of catalyst layers prepared with (**a**) Pd/MnO_2_, (**b**) Pd/C, and (**c**) Pd/MnO_2_:Pd/C.

Figure 8 shows cross-sectional SEM micrographs of the catalytic layers corresponding to Pd/MnO_2,_ Pd/C, and the Pd/MnO_2_:Pd/C hybrid. Marked differences in the thickness and architecture of the catalytic layer are observed depending on the support used.

The Pd/C-based layer exhibits a reduced average thickness (3.54 ± 0.88 µm), consistent with the high compaction typical of layers dominated by carbon black. In contrast, Pd/MnO_2_ exhibits an intermediate thickness (7.58 ± 2.84 µm), associated with the rigid and particulate morphology of the oxide.

Notably, the Pd/MnO_2_:Pd/C hybrid catalyst develops a significantly thicker catalytic layer (38.74 ± 3.45 µm), with a more open and heterogeneous texture. This substantial increase in thickness does not imply a simple accumulation of material, since identical catalyst loadings were employed, but rather reflects the formation of a more porous and less compact architecture, where MnO_2_ particles act as physical spacers between carbon-rich and Pd-rich domains.

This morphology favors the generation of extended channels for the transport of gaseous reactants (H_2_ and O_2_), the evacuation of products, and the redistribution of water within the catalytic layer. In this context, MnO_2_ fulfills a structural function as a spacer, mitigating excessive carbon compaction and preserving the accessibility of Pd active sites.

The more open architecture observed in the hybrid provides a structural basis consistent with the improved electrochemical performance and enhanced stability observed in both half-cell and full AEMFC tests, particularly under high-current-density conditions where mass transport limitations become critical.

### 3.4. Comparative Performance Analysis

The electrocatalytic performance of the Pd/MnO_2_:Pd/C (40:60) hybrid catalyst compares favorably with various Pd- and MnO_2_-based systems previously reported for the HOR and ORR in AEMFCs, as summarized in Table 5. Under moderate operating conditions (60 °C, 5 cm^2^ cell area, 0.125 L/min H_2_, 0.250 L/min O_2_, the hybrid catalyst used at the cathode achieved a current density of 205.4 mA cm^−2^ at 0.6 V and a peak power density of 128.0 mW cm^−2^. These values surpass those reported for MnO_2_-based cathodes such as Pd@MnO_2_ (100 mA cm^−2^, 66 mW cm^−2^) [22], MnO_2_@400 °C (125 mA cm^−2^, 82 mW cm^−2^) [30], and α-MnO_2_/C (57 mA cm^−2^, 45.2 mW cm^−2^) [31], despite the fact that these systems often operated under higher temperatures, increased gas flow rates, or more elaborate support morphologies.

Notably, the Pd/MnO_2_:Pd/C hybrid was synthesized through a simple physical mixing approach, without the use of surfactants, dopants, or chemical anchoring strategies, effectively integrating the redox activity of MnO_2_ with the high conductivity and dispersion capacity of carbon black. This straightforward methodology resulted in enhanced catalyst accessibility and stability.

When employed as the anodic material in combination with a commercial Pt/C cathode, the same hybrid catalyst delivered a current density of 296.3 mA cm^−2^ at 0.6 V and a peak power density of 221.7 mW cm^−2^. This performance exceeds that of several Pt/C–Pt/C configurations reported under comparable or even more favorable conditions, which typically yield power densities between 109 and 188.5 mW cm^−2^ and current densities of 222.7–250.0 mA cm^−2^ at 0.6 V [32,33]. It also compares well with other Pd-based bifunctional systems, including Ag-alloyed Pd catalysts (180.0 mW cm^−2^ at 73 °C, 1.0 L/min air flow) [34] and PdNi alloys (372.0 mA cm^−2^ at 0.6 V) [26], particularly when considering the more conservative operating conditions and lower material costs associated with this work.

These findings highlight that the synergistic integration of Pd/MnO_2_ and Pd/C enables competitive AEMFC performance without relying on high-temperature treatments, surfactant-assisted synthesis, or costly alloying elements. Beyond its catalytic contribution, MnO_2_ serves a dual function as both a structural and stabilizing phase, fostering a porous electrode architecture and contributing to the mechanical and electrochemical durability of the hybrid catalyst.

## 4. Conclusions

This work demonstrates the effectiveness of Pd-based electrocatalysts supported on β-MnO_2_, Vulcan carbon, and their 40:60 hybrid (Pd/MnO_2_:Pd/C) as bifunctional materials for AEMFCs. The catalysts showed uniform Pd nanoparticle distribution, with Pd/C exhibiting the highest surface area (51.5 m^2^ g^−1^) and Pd/MnO_2_ the lowest (16.1 m^2^ g^−1^), while the hybrid maintained an intermediate value (37.0 m^2^ g^−1^), indicating effective physical blending.

Electrochemical tests revealed that the hybrid outperformed the individual components for both the ORR and HOR. It exhibited the lowest Tafel slope for the ORR (60.9 mV dec^−1^) and the highest exchange current density (8.59 × 10^−5^ mA cm^−2^), as well as improved HOR kinetics (Tafel slope of 35.0 mV dec^−1^, J_0_ = 5.68 × 10^−5^ mA cm^−2^). CO stripping analysis further showed that the Pd/MnO_2_:Pd/C hybrid possesses the highest electrochemically active surface area, enabling superior intrinsic (mass-normalized) activity toward both reactions and evidencing a more efficient utilization of Pd active sites.

In AEMFCs, the Pd/MnO_2_:Pd/C catalyst achieved peak power densities of 128.0 mW cm^−2^ as a cathode and 221.7 mW cm^−2^ as an anode, exceeding the performance of the Pd/C and Pd/MnO_2_ catalysts.

Moreover, accelerated durability tests showed exceptional stability, with the hybrid retaining 100% of its ORR and 99% of its HOR activity after 3000 cycles, in contrast to the 68–70% retention rate observed for Pd/C. These results confirm the synergistic effect of combining MnO_2_ and carbon, where MnO_2_ acts as a catalytic and structural enhancer, improving activity, mass transport, and long-term durability. The Pd/MnO_2_:Pd/C catalyst thus represents a promising reduced-PGM-content alternative for high-performance and durable AEMFC electrodes.

## Figures and Tables

**Table 1:** Specific surface area, pore diameter, pore size, and Pd content of catalysts.

Catalyst	Specific Surface (m^2^ g^−1^)	Pore Volume (cm^3^ g^−1^)	Pore Size (nm)	Pd (%w)
Pd/MnO_2_:Pd/C	37.01	2.24 × 10^−2^	1.78	10.0 ± 0.5
Pd/C	51.50	9.26 × 10^−2^	1.76	9.5 ± 0.7
Pd/MnO_2_	16.10	9.58 × 10^−3^	1.80	9.6 ± 0.4

**Table 2:** Catalytic activity and kinetic parameters obtained from electrocatalysts for ORR.

Catalyst	E_1/2_ (V vs. RHE)	E_−1.5 mA cm^−2^_ (V vs. RHE)	J_0.2 V vs. RHE_ (mA cm^−2^)	Tafel Slope (mV dec^−1^)	*J*_0_ (mA cm^−2^)
Pd/MnO_2_:Pd/C	0.79	0.81	−5.88	60.9	8.59 × 10^−5^
Pd/C	0.73	0.74	−3.67	73.6	1.89 × 10^−5^
Pd/MnO_2_	0.71	0.32	−1.57	82.6	1.09 × 10^−6^

**Table 3:** Catalytic activity and kinetic parameters obtained from electrocatalysts for HOR.

Catalyst	E_1/2_ (V vs. RHE)	E_0.5 mA cm^−2^_ (V vs. RHE)	J_0.3 V vs. RHE_ (mA cm^−2^)	Tafel Slope (mV dec^−1^)	*J*_0_ (mA cm^−2^)
Pd/MnO_2_:Pd/C	0.21	0.13	2.33	35.0	5.68 × 10^−5^
Pd/C	0.30	0.22	1.04	41.2	2.91 × 10^−5^
Pd/MnO_2_	0.39	0.54	0.13	35.1	9.21 × 10^−6^

**Table 5:** Fuel cell performance parameters of different materials in anodes and cathodes.

Anode	Cathode	*J*_0.6 V_ ($mA\;{\mathrm{cm}}^{-2}$)	P_max_ ($mW\;{\mathrm{cm}}^{-2}$)	Test Conditions	Ref.
Pt/C	Pd/MnO_2_:Pd/C	205.4	128.0	60 °C, H_2_: 0.125 L min^−1^, O_2_: 0.250 L min^−1^, Membrane: AF1-HNN8-25-X, Aemion™	This work
Pd/MnO_2_:Pd/C	Pt/C	296.3	221.7	60 °C, H_2_: 0.125 L min^−1^, O_2_: 0.250 L min^−1^, Membrane: AF1-HNN8-25-X, Aemion™	This work
Pt/C	Pd@MnO_2_	100.0	66.0	60 °C, H_2_: 0.200 L min^−1^, O_2_: 0.200 L min^−1^, Membrane: not specified	[22]
Pt/C	MnO_2_@400 °C	125.0	82.0	50 °C, H_2_: 0.200 L min^−1^, O_2_: 0.300 L min^−1^, Membrane: not specified	[29]
Pt/C	α-MnO_2_/C	57.0	45.2	50 °C, H_2_: 0.200 L min^−1^, O_2_: 0.300 L min^−1^, Membrane: FAA-3	[30]
Pt/C	Pt/C	101.3	61.8	50 °C, H_2_: 0.200 L min^−1^, O_2_: 0.300 L min^−1^, Membrane: FAA-3	[30]
Pt/C	Mn_x_O_y_-GC	135.0	98.0	70 °C, H_2_: 0.200 L min^−1^, O_2_: 0.200 L min^−1^, Membrane: FAA-3	[31]
Pt/C	Pt/C	222.7	188.5	70 °C, H_2_: 0.100 L min^−1^, O_2_: 0.100 L min^−1^, Membrane: FAA-3	[31]
Pt/C	Pt/C	250.0	109.0	60 °C, H_2_: 0.100 L min^−1^, O_2_: 0.100 L min^−1^, Membrane: not specified	[32]
Pd/C	Ag alloy	80.0	180.0	73 °C, H_2_: 0.200 L min^−1^, O_2_: 1.000 L min^−1^, Membrane: not specified	[33]
PdNi/C	Pt/C	372.0	262.0	60 °C, H_2_: 0.400 L min^−1^, O_2_: 0.800 L min^−1^, Membrane: AF1-HNN8-25-X, Aemion™	[25]

**Table 4:** A summary of the ECSA results of the Pd/MnO_2_:Pd/C blend and pristine Pd/C and Pd/MnO_2_ electrocatalysts after CO stripping.

Catalyst	Q_CO_ (μC)	ECSA (m^2^ g^−1^)
Pd/MnO_2_:Pd/C	48.41	20.17
Pd/C	32.09	13.37
Pd/MnO_2_	8.15	3.40

## Data Availability

The data presented in this study are available om corresponding author.

## Supplementary Materials

The following supporting information can be downloaded at: https://www.mdpi.com/article/10.3390/nano16010071/s1, Figure S1: Influence of the Pd/MnO_2_:Pd/C mass ratio on the electrochemical activity toward (a) ORR and (b) HOR in alkaline medium. Figure S2: Koutecky–Levich plots for ORR on Pd/MnO_2_, Pd/C, and Pd/MnO_2_:Pd/C electrocatalysts.
