# Supplementary material for: Pd/MnO2:Pd/C Electrocatalysts for Efficient Hydrogen and Oxygen Electrode Reactions in AEMFCs
Source: Nanomaterials (Basel). 2026 Jan 4;16(1):71. doi: 10.3390/nano16010071 (PMC12787392; doi:10.3390/nano16010071)
Supplement: Supplementary file 1 [file nanomaterials-16-00071-s001.zip › nanomaterials-4053808-supplementary.docx]

Supplementary Information

**Pd/MnO_2_ : Pd/C Electrocatalysts for Efficient Hydrogen and Oxygen Electrode Reactions in AEMFCs**

**Ivan Cruz-Reyes,^1^ Balter Trujillo-Navarrete,^1,^* Moisés Israel Salazar-Gastélum,^1^ José Roberto Flores-Hernández,^2^ Tatiana Romero-Castañón,^2^ Rosa María Félix-Navarro^1,^***

^1^ Tecnológico Nacional de México/Instituto Tecnológico de Tijuana/Centro de Graduados e Investigación en Química. Blvd. Alberto Limón Padilla S/N, Mesa de Otay. C.P. 22000 Tijuana, Baja California, México. ivan.cruz19@tectijuana.edu.mx (I.C.R.); balter.trujillo@tectijuana.edu.mx (B.T.-N.); moises.salazar@tectijuana.edu.mx (M.S.-G.); rmfelix@tectijuana.mx (R.M.F.-N.);

^2^ Instituto Nacional de Electricidad y Energías Limpias (INEEL). Ave. Reforma 113, Col. Palmira, C.P. 62490. Cuernavaca, Morelos, México. jrflores@ineel.mx (J.R.F.-H); tromero@ineel.mx (T.R.-C.)

***** Correspondence: rmfelix@tectijuana.mx; balter.trujillo@tectijuana.edu.mx Tel.: +52 6646234043

**Ratio Effect of Pd/MnO_2_:Pd/C.** The influence of the Pd/MnO_2_:Pd/C mass ratio on the oxygen reduction reaction (ORR) in 0.1 M NaOH was systematically investigated to determine the optimal catalyst composition. Figure S1a compares the polarization curves of Pd/MnO_2_, Pd/C, and their physical mixtures at different ratios, clearly illustrating that combining both materials substantially alters the electrochemical response.

The pristine Pd/MnO_2_ catalyst exhibits limited activity, delivering a current density of only −1.57 mA cm^-2^ at 0.2 V vs RHE and requiring a low potential of 0.32 V vs RHE to reach −1.5 mA cm^-2^, indicative of poor intrinsic ORR kinetics. In contrast, commercial Pd/C demonstrates superior performance, achieving −3.67 mA cm^-2^ at 0.2 V vs RHE and an onset potential of 0.74 V vs RHE at −1.5 mA cm^-2^. Remarkably, the mixed Pd/MnO_2_:Pd/C catalysts display a pronounced synergistic effect. Among all tested compositions, the 40:60 ratio provides the highest activity, reaching −5.88 mA cm^-2^ at 0.2 V vs RHE and a value of 0.81 V vs RHE at −1.5 mA cm^-2^. This represents a significant enhancement compared to either single-component catalyst. Similar improvements are observed for the 50:50 and 30:70 ratios, although with slightly lower current densities. Conversely, further dilution of Pd/MnO_2_ (20:80) yields a response increasingly similar to Pd/C, suggesting that the synergistic contribution of MnO_2_ diminishes at high Pd/C contents.

The effect of the Pd/MnO_2_:Pd/C ratio on the hydrogen oxidation reaction (HOR) was also evaluated under the same alkaline conditions (0.1 M NaOH), as shown in Figure S1b. The pristine Pd/MnO_2_ catalyst exhibits poor HOR activity, with a current density of only 0.13 mA cm^-2^ at 0.3 V vs RHE and requiring 0.54 V vs RHE to reach 0.5 mA cm^-2^. In contrast, Pd/C achieves 1.04 mA cm^-2^ at 0.3 V vs RHE and requires only 0.22 V vs RHE to reach 0.5 mA cm^-2^. Notably, the blended catalysts again show clear synergistic enhancement, with the 40:60 composition delivering the best HOR performance: a current density of 2.33 mA cm^-2^ at 0.3 V vs RHE and the lowest potential (0.13 V vs RHE) to reach 0.5 mA cm^-2^. Intermediate ratios (50:50 and 30:70) also outperform the single-component catalysts, whereas further dilution of Pd/MnO_2_ (20:80) reduces activity, approaching the behavior of Pd/C.

Overall, these results highlight that an optimal balance between Pd/MnO_2_ and Pd/C is essential to maximize both ORR and HOR kinetics. The observed enhancements likely arise from the combined effects of improved hydrogen adsorption on Pd sites and facilitated charge transfer through the conductive carbon support.


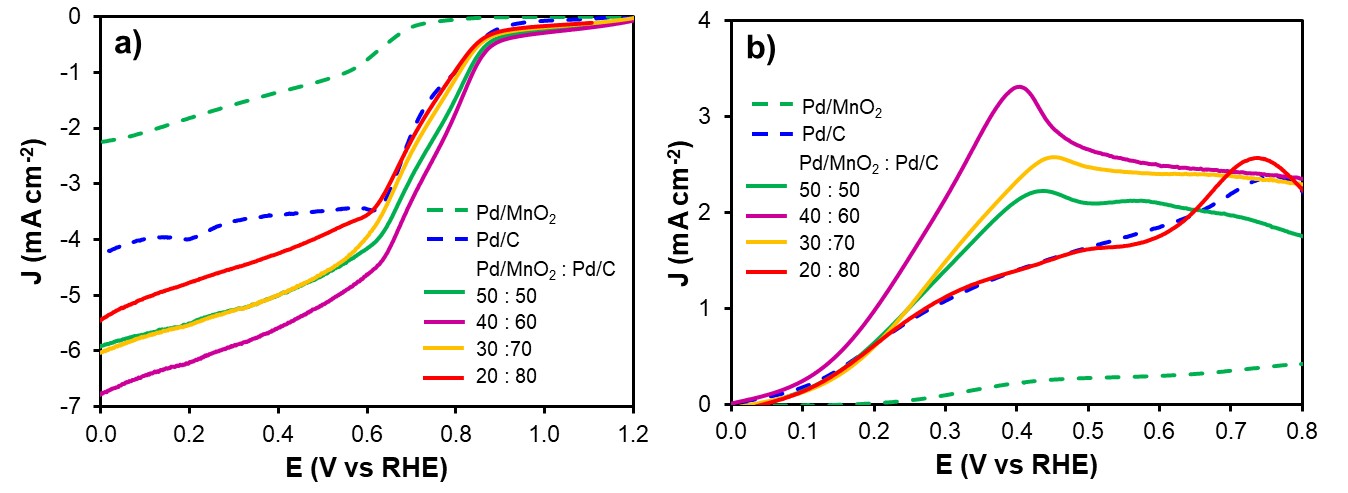


**Figure S1.** Influence of the Pd/MnO_2_:Pd/C mass ratio on the electrochemical activity toward (a) ORR and (b) HOR in alkaline medium.

**Koutecky–Levich Analysis of ORR.** The ORR kinetics of the Pd/MnO_2_, Pd/C, and Pd/MnO_2_:Pd/C electrocatalysts were further analyzed using the Koutecky–Levich (K–L) approach, based on the linear sweep voltammograms recorded at different rotation rates (Figure 3a–c) in O₂-saturated 0.1 M NaOH. The measured current densities were analyzed according to the K–L equation:

$$\frac{1}{J}=\frac{1}{J_{k}}+\frac{1}{B \omega^{\frac{1}{2}}}$$

where $J$ is the measured current density, $J_{k}$ is the kinetic current density, $\omega$ is the angular rotation rate, and $B$ is a constant related to the number of electrons transferred during the ORR.

The corresponding K–L plots (Figure S2) exhibit good linearity over the investigated rotation range, indicating first-order reaction kinetics with respect to dissolved oxygen and validating the applicability of the K–L model. By comparing the experimental slopes with the theoretical K–L lines corresponding to two- and four-electron transfer pathways, the average number of electrons transferred ($n$) was estimated.

The calculated $n$ values are approximately 3.6 for Pd/MnO_2_, 4.1 for Pd/C, and 4.0 for the Pd/MnO_2_:Pd/C hybrid catalyst. The value obtained for Pd/MnO_2_ suggests a mixed ORR pathway involving both two- and four-electron processes, consistent with the known tendency of MnO_2_-based materials to partially promote peroxide formation in alkaline media. In contrast, Pd/C exhibits an $n$value slightly above four, which may arise from experimental uncertainties or deviations from ideal mass transport behavior, but overall indicates a predominantly four-electron ORR pathway.

Notably, the Pd/MnO_2_:Pd/C hybrid catalyst shows an electron transfer number very close to four, demonstrating a highly selective four-electron ORR mechanism. This behavior suggests that the incorporation of Pd/C into the hybrid system effectively suppresses the formation of peroxide intermediates while maintaining the beneficial interaction between Pd and MnO_2_. These results are in good agreement with the enhanced ORR activity and favorable kinetic parameters discussed in Section 3.2.1, further supporting the synergistic role of the hybrid catalyst in promoting efficient oxygen reduction in alkaline media.

Based on the electron transfer numbers obtained from the Koutecky–Levich analysis, the ORR pathway can be correlated with the reaction schemes described in the Introduction. The Pd/MnO_2_ catalyst (n ≈ 3.6) follows a mixed pathway involving both the two-electron and four-electron processes (Reactions 2 and 1, respectively). In contrast, Pd/C (n ≈ 4.1) and the Pd/MnO_2_:Pd/C hybrid catalyst (n ≈ 4.0) predominantly proceed via the four-electron pathway (Reaction 1), which is desirable for fuel cell applications due to minimized peroxide formation.


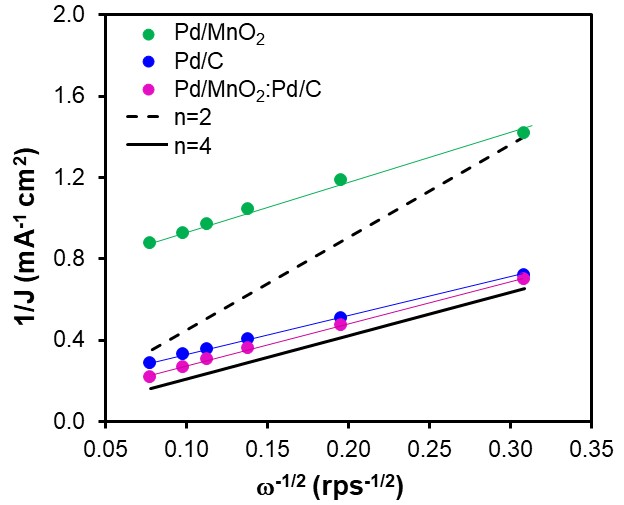


**Figure S2.** Koutecky–Levich plots for ORR on Pd/MnO_2_, Pd/C, and Pd/MnO_2_:Pd/C electrocatalysts.
